# The Magnitude and Effects of Early Integration of Palliative Care Into Oncology Service Among Adult Advanced Cancer Patients at a Tertiary Care Hospital

**DOI:** 10.7759/cureus.15313

**Published:** 2021-05-29

**Authors:** Elham H Ghabashi, Belal M Sharaf, Waheed A Kalaktawi, Retaj Calacattawi, Abdullah W Calacattawi

**Affiliations:** 1 Palliative Care, Ministry of Health, Jeddah, SAU; 2 Oncology, King Faisal Specialist Hospital and Research Center, Jeddah, SAU; 3 Family Medicine, Ministry of Health, Jeddah, SAU; 4 Medicine, King Saud Bin Abdulaziz University for Health Sciences, Jeddah, SAU; 5 Family Medicine, King Abdulaziz University Faculty of Medicine, Jeddah, SAU

**Keywords:** palliative care, consultation, referral, cancer, saudi arabia

## Abstract

Background

Palliative care (PC) has a positive effect on symptom burden, quality of life, psychosocial communication, prognostic understanding, mood, and quality of care at the end of life of patients with advanced cancer.

Objectives

To investigate the timing of the first palliative consultation and referral of advanced cancer patients to the palliative care service and their determinants at King Faisal Specialist Hospital and Research Center (KFSHRC), Jeddah, Saudi Arabia.

Subjects and methods

A retrospective cohort study was conducted at KFSHRC. It included advanced cancer patients who died between January 1, 2019 and Jun 30, 2020. The dependent variable of primary interest is the timing of PC consultation and the timing of PC referral. The independent variables included age, sex, marital status, nationality, date of death, types of cancer, Eastern Cooperative Oncology Group (ECOG), palliative performance status (PPS), palliative prognostic index (PPI), code status (do not resuscitate [DNR]), the severity of symptoms (assessed by the Edmonton Symptom Assessment System - Revised [ESAS-r]), referral to home health care (HHC), referral to long-term care (LTC), referral to interdisciplinary team (IDT), length of survival after the first PC consultation, length of survival after the referral to the PC service, length of hospital stay, frequency of emergency room (ER) visits and hospital admission in the last year before death, and involvement in bereavement with advanced care planning (ACP) services.

Results

Of the 210 advanced cancer patients, 109 (51.9%) were male, and their ages ranged between 18 and 90 years. More than half of patients (56.7%) had a history of PC consultation. Among them, PC consultation was described as late in 60.5% of patients. Concerning the timing of palliative care referral among advanced cancer patients, it was too late and much too late among 25.7% and 58.1% of them, respectively. Patients who visited ER more frequently (≥3 times) (p=0.014) and those who referred to HHC (p=0.005) were more likely to consult PC early compared to their counterparts. Length of survival was significantly higher among patients who reported early PC consultation compared to those without PC consultation and those with late PC consultation, p<0.001. Referral to PC for both transfer of care and symptom management was associated with earlier PC consultation, p=0.021. Patients who were admitted to the hospital three times or more were less likely to be much too late referred to PC services, p=0.046. Also, patients who were not referred to long-term care or home health care were more likely to be referred to PC services much too late, p<0.001. Among 28.8% of patients whose PPS ranged between 30% and 50% compared to 14.9% of those whose PPS ranged between 10% and 20% expressed too late referral time to PC, p=0.040.

Conclusion

In a considerable proportion of terminal cancer patients, palliative care was consulted late, and the timing of palliative care referral was too late/much too late among most of those consulted palliative care. Length of survival was higher among patients who reported early PC consultation and who with ideal referral time to PC services than others. Therefore, future considerations to facilitate early integration of palliative care in cancer patients are highly recommended through mainly improving staff education in communication skills and palliative care approach.

## Introduction

World Health Organization (WHO) defines palliative care (PC) as “the prevention and relief of suffering of adult and pediatric patients and their families facing the problems associated with life-threatening illness” [1]. Palliative care aims to improve the quality of life and quality of care of patients with advanced and life-threatening illnesses by approaching physical, psychosocial, and spiritual symptoms and concerns [2].

Palliative care has a positive effect on symptom burden, quality of life, psychosocial communication, prognostic understanding, and decision making, mood, satisfaction with the care received, and quality of care at the end of life [3].

However, WHO has been advocating for the early application of palliative care principles in the management of chronic life-threatening conditions including incurable cancer [4]. Also, several studies support the concept of the early introduction of palliative care into patients since the first time of diagnosis of cancer to improve the outcomes [5-7].

Additionally, early palliative care was supported by the American Society of Clinical Oncology [8]. Furthermore, numerous randomized controlled trials support the involvement of palliative care within eight weeks of diagnosis of advanced cancer to improve quality of life and outcomes [5].

Recent evidence suggests that regardless of the referral timing, palliative care consultation may be associated with some benefits such as the decreased likelihood of unnecessarily aggressive interventions in terminally ill cancer patients [9].

Despite the growing evidence emphasizing the multifaceted benefits of adopting this approach of early integration of palliative care in the management of advanced cancer, delayed initiation of palliative care referral continues to be a common concern, with a significant portion of patients with advanced cancer still dying with no access to PC services [10].

This study aims to estimate the magnitude of early integration of palliative care service into oncology service among advanced cancer patients at King Faisal Specialist Hospital and Research Center (KFSHRC), Jeddah, Saudi Arabia.

## Materials and methods

This study involved a retrospective longitudinal analytic cohort by review of patients’ medical records during the period between January 1, 2019 and Jun 30, 2020.

It was conducted and approved at KFSHRC, which is one of the main referral centers for public cancer treatment in Saudi Arabia. The hospital has a specific adult oncology unit with 30 hospital beds (15 beds for adult oncology, palliative care, and 15 beds for adult oncology hematology), a dedicated multi-professional PC team, inpatient and outpatient PC units, and a home care service. The PC unit focuses on providing comprehensive patient care from the early outpatient management of symptoms to end-of-life care and supporting family and loved ones throughout the disease process. The referral of patients to PC is performed by the medical team responsible for the standard oncology treatment independent of the specialty from both inpatient and outpatient general oncology units.

The dependent variable of primary interest was PC consultation (PCC) and its timing, which was divided into two categories concerning the date of death: early consultation (≥3 months between first PC consultation and death) and late consultation (<3 months between first PC consultation and death) [11]. The second one was PC referral and its timing, which was arbitrarily divided into five categories: much too late (<1 month), too late (1 to 3 months), appropriate (3 to 6 months), appropriate/ideal (6 to 24 months), much too early (>24 months) concerning the date of death. Both dependent variables were identified by including all sociodemographic and clinical variables [12].

The independent variables such as age, sex, marital status, nationality, date of death, types of cancer, the severity of symptoms (assessed by ESAS-r), an Eastern Cooperative Oncology Group (ECOG), palliative performance status (PPS), palliative prognostic index (PPI), code status (do not resuscitate [DNR]), referral to home health care (HHC), referral to long term care (LTC), referral to interdisciplinary team (IDT), length of survival after the first consultation of the PC service, length of survival after the referral to the PC service, length of hospital stay (LOS), frequency of emergency room (ER) visits in the last year before death, frequency hospital admission in the last year before death, and involvement in advanced care planning (ACP) with bereavement services.

Terms and definitions

Length of hospital stay is the difference between the last admission date and discharge date (death/HHC/LTC). Length of survival is the difference between referral date and death date. Do Not Resuscitate (DNR) is the consent that should be taken in print (hard copy) or verbally from patients or their caregivers and documented in the medical records. Subjects in this study were classified into three groups according to the time interval between signing DNR and death: within 3 days before death, 4 days to 2 weeks, and >2 weeks. Palliative performance status (PPS) is concerned with the functional status, including ambulation, the extent of the disease, activity, self-care, oral intake, and consciousness level. It is a predictor of survival as PPS 10-20 is equivalent to a median survival of 6 days, 30-50 equivalents to 41 median survival, and 60-70 equivalent to a median survival of 108 days [13]. Eastern Cooperative Oncology Group (ECOG) indicates the patient's performance status and is predictive for survival and applied in the selection of patients for chemotherapy [13]. Palliative prognostic index (PPI) is a prognostic scale and includes PPS, dyspnea, edema, oral intake, and delirium, which can be applied to patients with cancer, whether in the hospital or at home. According to its score, patients were assigned into three groups: A - survival times more than 6 weeks, B - 3-6 weeks, or C - less than 6 weeks [13].

The inclusion criteria include all patients with advanced cancer who died between January 1, 2019 and Jun 30, 2020. 210 cancer patients were part of the sample of the study irrespective of nationality and gender, provided that they were older than 18 years. The code status for the patients was either full code at the time of first PC consultation or DNR declared at the time of referral to PC services. Eligibility of patients: patients needed to have at least 3 months of clinical follow-up in the hospital immediately before death. Advanced cancer was determined as any type of cancer that was either curable or incurable or metastatic, including hematologic malignancy, or cases of solid tumors and recurrent inoperable disease. Patients with an Eastern Cooperative Oncology Group (ECOG) or palliative performance status (PPS): exclusion criteria were non-cancer patients, patients under 18 years old, patients in ICU on ventilators, and patients who had been screened already in the pilot study.

Institutional Review Board approval was obtained for the study: IRB number 2020-80.

Data were obtained from patient records utilizing a medical record composed of three parts: first, sociodemographic data (age, sex, nationality, and date of death); second, clinical data (types of cancer, the severity of symptoms, prognostic indicators [ECOG, PPS, PPI], and quality of care); third, data on the consultation and referral to PC (PC consultation with the date of the first consultation and referral to PC with the date of referral).

Data were entered into a personal computer by the researcher using SPSS program version 25 (IBM Corp., Armonk, NY, USA). Categorized variables were described using frequency and percentage whereas continuous quantitative variables were described using an arithmetic mean, range, and standard deviation (SD). Estimation and inference were made by Chi-squared and ANOVA tests, and a p-value of <0.05 was considered statistically significant.

## Results

The study group comprised only Saudi nationals. Other demographic and basic characteristics are summarized in Table 1. Males represent 51.9% of them. Their age ranged between 18 and 90 years with a mean±SD of 61.4±14.4 years. The majority of them (79.5%) were married. Eligibility for palliative care was observed in 83.8% of patients. Concerning the type of cancer, the gastrointestinal tract ranked first, either upper (21%) or lower (20.5%), followed by breast (11.9%) and head and neck cancers (11.4%) (Table 1).

**Table 1:** Demographic and basic characteristics of patients in the study group. GIT: gastrointestinal tract

Variables		Frequency	Percentage (%)
Gender	Male	109	51.9
	Female	101	48.1
Age (years)	Range	18-90	
	mean±SD	61.4±14.4	
Marital status	Single	12	5.7
	Married	137	79.5
	Divorced	5	2.4
	Widowed	26	12.4
Eligibility for palliative care	Yes	176	83.8
	No	34	16.2
Type of cancer	Gynecological	20	9.5
	Upper GIT	44	21.0
	Lower GIT	43	20.5
	Breast	25	11.9
	Hematological	8	3.8
	Head and neck	24	11.4
	Respiratory	20	9.5
	Urological	21	10.0
	Others	5	2.4

Palliative care consultation

**Figure 1:**
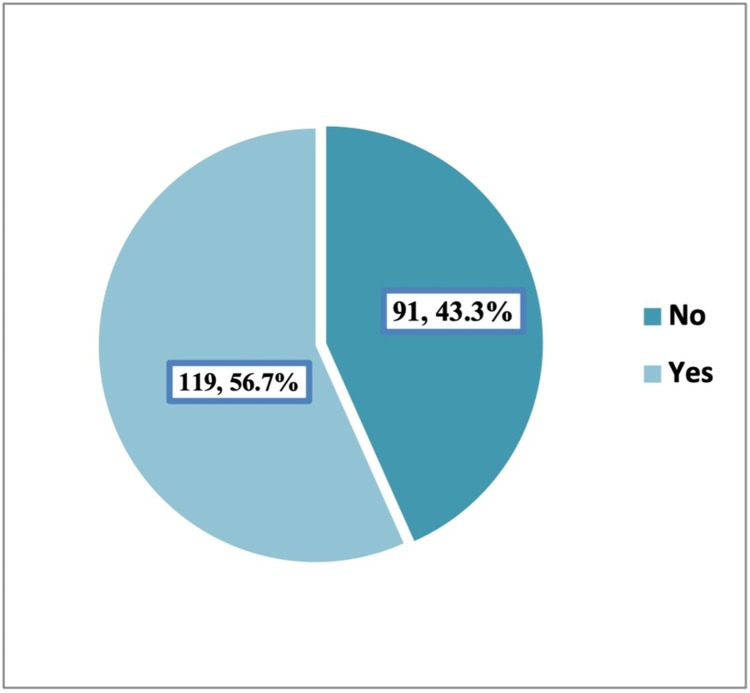
The prevalence of palliative care consultation among advanced cancer patients. Yes: palliative care consulted; No: no palliative care consulted

**Figure 2:**
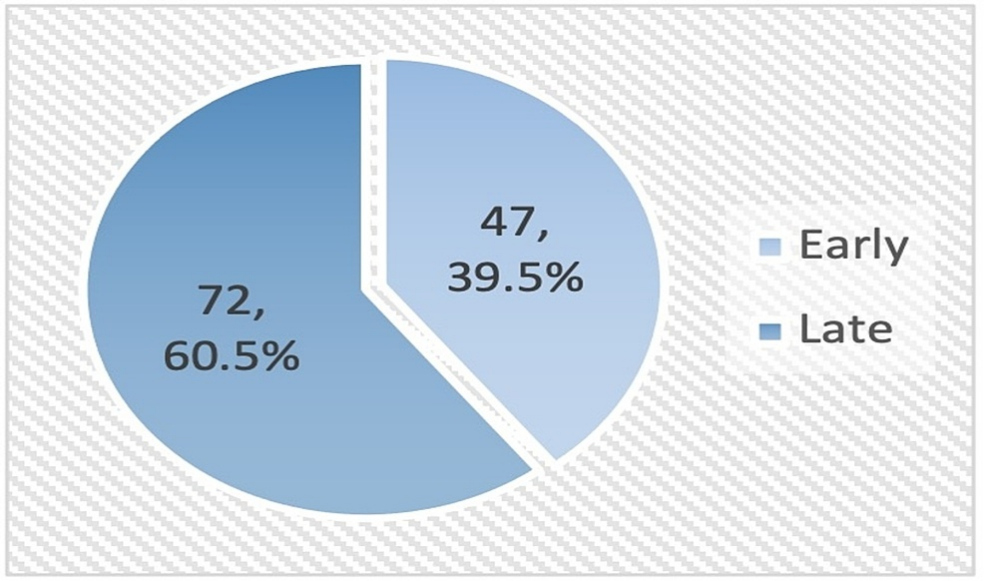
Timing of palliative care consultation among advanced cancer patients who sought consultation. Early consultation: ≥3 months between first PC consultation and death; late consultation: <3 months between first PC consultation and death

More than half of the patients (56.7%) had a history of PC consultation (Figure 1). Among them, PC consultation was described as late in 60.5% of patients (Figure 2). None of the studied demographic/basic characteristics of the patients (age, gender, marital status, eligibility, and type of cancer) was significantly associated with PC consultation time. Almost a third (30.1%) of patients who visited ER more frequently (≥3 times) compared to only 13.4% of those who visited ER less frequently (0-2 times) expressed early PC consultation, p=0.014. Patients who were referred to home health care were more likely to consult PC early compared to their counterparts, p=0.005. Length of survival was significantly higher among patients who reported early PC consultation compared to those without PC consultation and those with late PC consultation (101.7±129.4 versus 59.8±100.1 and 22.9±44.8, respectively), p<0.001. Referral to PC for both transfer of care and symptom management was associated with earlier PC consultation, p=0.021 (Table 2). None of the studied prognostic factors was significantly associated with PC consultation time.

**Table 2:** Association between quality of care and quality of life with palliative care consultation time in patients with advanced cancer. ER: emergency room; PC: palliative care *Chi-square test; **ANOVA

Variables	PC consultation			P-value*
	No	Yes		
		Early	late	
	N=91(%)	N=47(%)	N=72(%)	
Frequency of hospital admission				0.071
0-2 (n=84)	40 (47.6)	12 (14.3)	32 (38.1)	
≥3 (n=126)	51 (40.5)	35 (27.8)	40 (31.7)	
Frequency of ER visits in the last year of life				0.014
0-2 (n=97)	48 (49.5)	13 (13.4)	36 (37.1)	
≥3 (n=113)	43 (38.1)	34 (30.1)	36 (31.9)	
Referral to Long Term Care				0.246
No (n=196)	83 (42.4)	43 (21.9)	70 (35.7)	
Yes (n=14)	8 (57.1)	4 (28.6)	2 (14.3)	
Referral to Home Health Care				0.005
No (n=90)	36 (40.0)	13 (14.4)	41 (45.6)	
Yes (n=120)	55 (45.9)	34 (28.3)	31 (25.8)	
Referral to Interdisciplinary Team				0.082
No (n=87)	60 (48.7)	28 (22.8)	25 (28.5)	
Yes (n=123)	31 (35.6)	19 (21.8)	37 (42.6)	
Length of Hospital Stay (days)				0.371**
Mean±SD	19.7±19.4	25.7±29.5	22.0±23.7	
Length of survival after first referral to PC services				<0.001**
Mean±SD	59.8±100.1	101.7±129.4	22.9±44.8	
Actively Dying				0.338*
Yes (n=33)	12 (36.4)	6 (18.2)	15 (45.5)	
No (n=177)	79 (44.6)	41 (23.2)	57 (32.2)	
Reason of referral				0.021
Transfer of care (n=97)	52 (53.6)	18 (18.6)	27 (27.8)	
Both of transfer of care and symptom management (n=113)	39 (34.5)	29 (25.7)	45 (39.8)	

Palliative care referral

**Figure 3:**
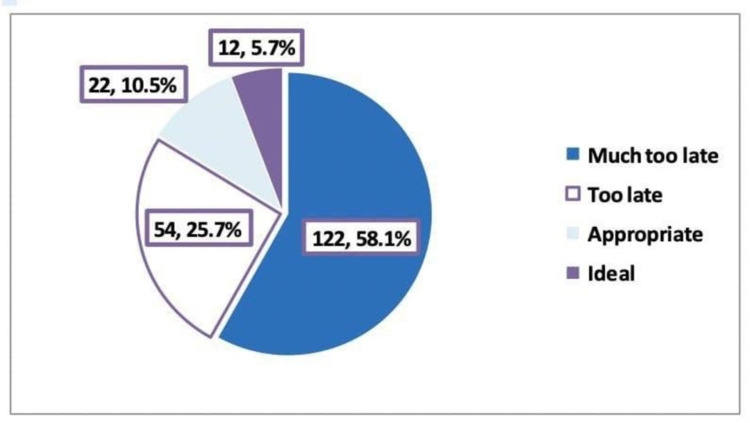
Timing of palliative care referral among advanced cancer patients. Much too late (<1 month), too late (1 to 3 months), appropriate (3 to 6 months), appropriate/ideal (6 to 24 months), much too early (>24 months) in relation to date of death.

Concerning the timing of palliative care referral among advanced cancer patients, it was too late and much too late among 25.7% and 58.1% of them, respectively (Figure 3). Severe not well-being (59.1%), tiredness (49%), loss of appetite (29.5%), and shortness of breath (18.6%) were the most frequently reported among patients with advanced cancer. No pain, drowsiness, nausea, depression, and anxiety were observed among 64.8%, 59%, 72%, 72.9%, and 72.3% of patients, respectively.

**Figure 4:**
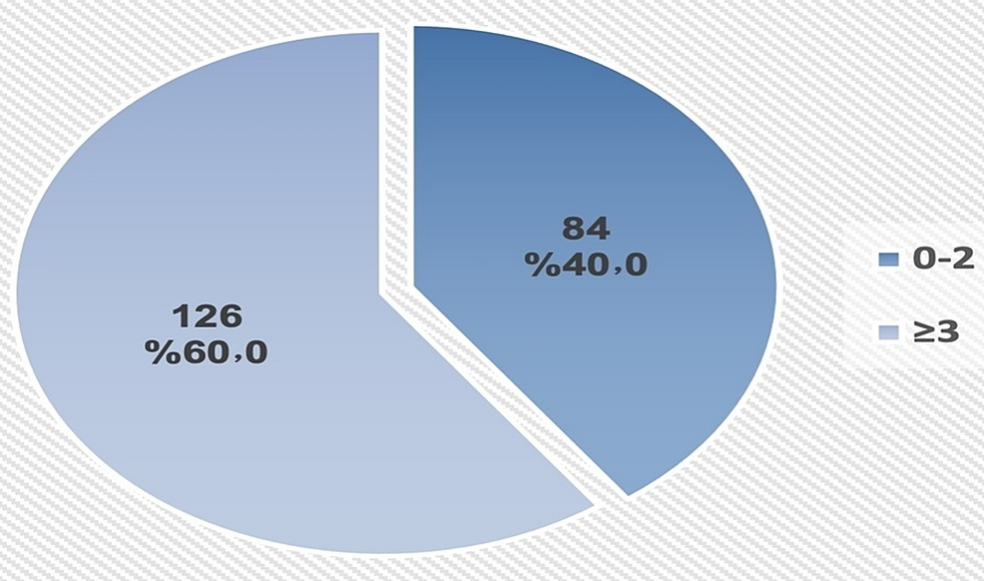
Frequency of hospital admission in the last year of life among patients with advanced cancer.

**Figure 5:**
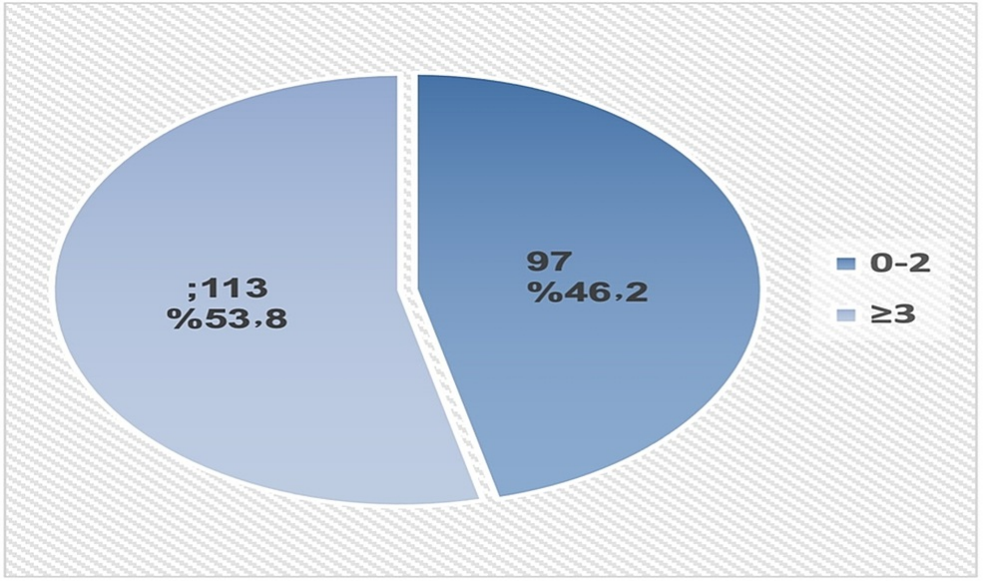
Frequency of emergency room visits in the last year of life among patients with advanced cancer.

As shown in Figure 4, 60% of the patients had three or more hospital admissions in last year of life, whereas 53.8% had three or more visits to the ER (Figure 5).

**Figure 6:**
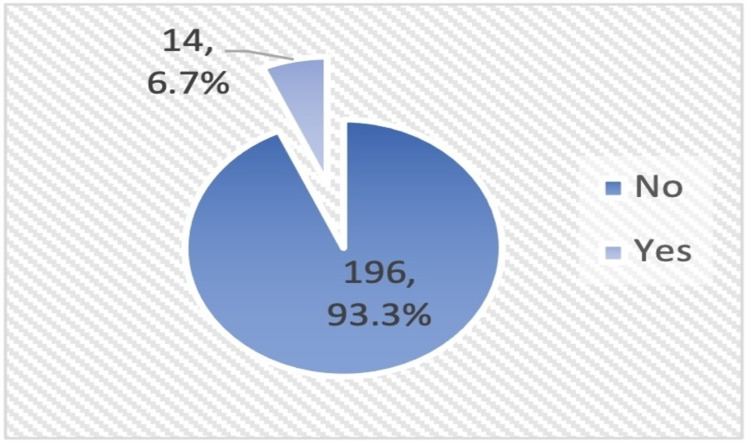
Prevalence of referral to long term care among patients with advanced cancer.

**Figure 7:**
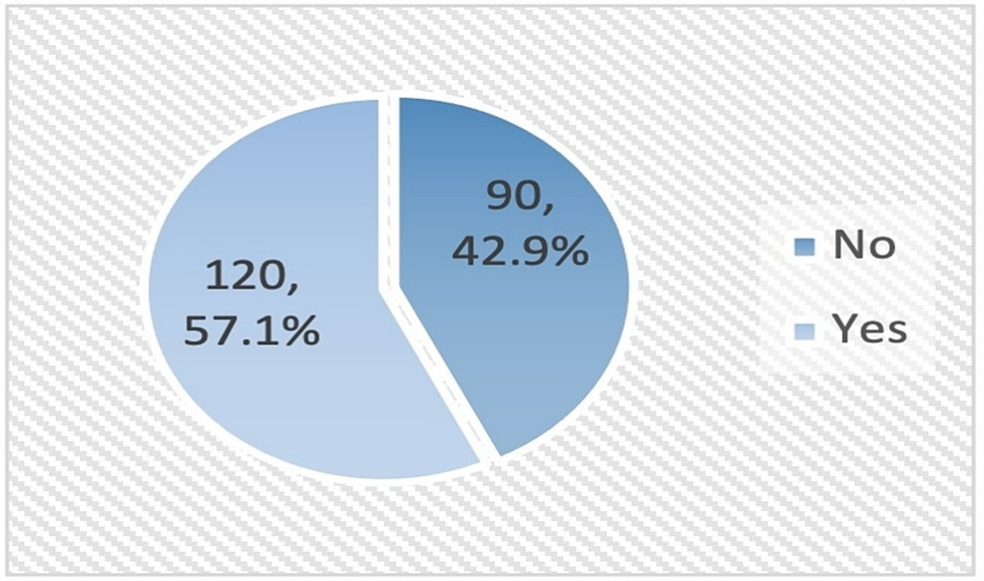
Prevalence of referral to home health care among patients with advanced cancer.

History of referral to long-term care was observed among a minority of patients with advanced cancer (6.7%) (Figure 6), while history of referral to home health care was reported among 57.1% of them (Figure 7).

**Figure 8:**
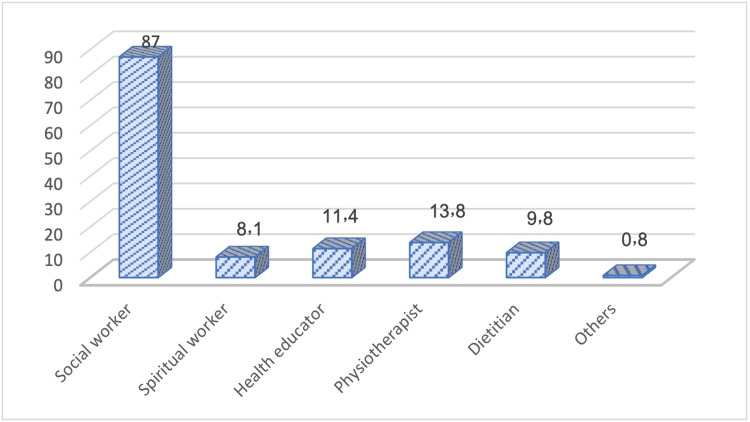
: Interdisciplinary team to whom patients with advanced cancer were referred.

More than half of the participants (58.6%) reported history of referral to interdisciplinary team. Among them, social workers ranked first (87%) (Figure 8).

**Figure 9:**
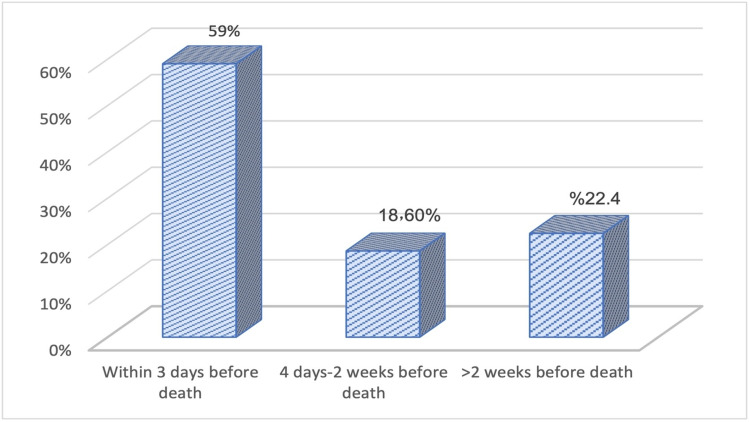
The time interval between signing the DNR order and the patient’s death. DNR: do not resuscitate

Regarding the time interval between signing the DNR order and the patient’s death, it was within three days before death in 59% of patients whereas it exceeded two weeks among 22.4% of them (Figure 9).

Length of hospital stay ranged between one and 153 days with a mean±SD of 21.8±23.5 days whereas the length of survival after first referral to PC services ranged between 0 and 663 days (56.5±97.6) and the median was 24 days. The reason for referral to PC services was mainly both transfer of care and symptom management (53.8%). Thirty-three patients (15.7%) were actively dying (Table 3).

**Table 3:** Quality of care and quality of life in patients with advanced cancer.

Variables	Description
Length of hospital stay (days)	
Range	1-153
Mean±SD	21.8±23.5
Length of survival after referral to palliative care services	
Range	0-663
Mean±SD	56.5±97.6
Median	24
Reason of referral	
Transfer of care	97 (46.2%)
Both (Transfer of care and symptom management)	113 (53.8%)
Actively dying	
Yes	33 (15.7%)
No	177 (84.3%)

Regarding prognostic factors, the majority of patients with advanced cancer belonged to category 4 of the ECOG (97.1%). Palliative performance status ranged between 30% and 50% in most of the participants (77.6%). Most of the participants belonged to group C (98.6%) according to PPI.

None of the studied demographic/basic characteristics of the patients (age, gender, marital status, eligibility, and type of cancer) was significantly associated with PC referral time.

Patients with no pain, no impact on wellbeing, no loss of appetite, and moderate depression were more likely than others to be referred for PC service much too late, p=0.008, 0.012, 0.011, and 0.023 respectively. Patients with severe nausea were less likely to be referred much too late to PC services (Table 4).

**Table 4:** Association between severity of the presenting symptoms and palliative care referral time among patients with advanced cancer. *Chi-square test

Severity of symptoms	Timing of palliative care referral				P-value*
	Much too late	Too late	Appropriate	Ideal	
	N=122 (%)	N=54 (%)	N=22 (%)	N=12 (%)	
Pain					
No (n=136)	91 (66.9)	27 (19.9)	12 (8.8)	6 (4.4)	0.008
Mild (n=10)	4 (40.0)	5 (50.0)	0 (0.0)	1 (10.0)	
Moderate (n=57)	23 (40.4)	20 (35.1)	9 (15.8)	5 (8.8)	
Severe (n=7)	4 (57.1)	2 (28.6)	1 (14.3)	0 (0.0)	
Tiredness					
No (n=9)	3 (33.3)	4 (44.4)	1 (11.1)	1 (11.1)	0.281
Mild (n=9)	7 (77.8)	2 (22.2)	0 (0.0)	0 (0.0)	
Moderate (n=89)	44 (49.4)	30 (33.7)	10 (11.2)	5 (5.6)	
Severe (n=103)	68 (66.0)	18 (17.5)	11 (10.7)	6 (5.8)	
Drowsiness					
No (n=124)	76 (61.3)	33 (26.6)	9 (7.3)	6 (4.8)	0.057
Mild (n=31)	12 (38.7)	14 (45.2)	4 (12.9)	1 (3.2)	
Moderate (n=46)	28 (60.9)	7 (15.2)	7 (15.2)	4 (8.7)	
Severe (n=9)	6 (66.7)	0 (0.0)	2 (22.2)	1 (11.1)	
Nausea					
No (n=151)	93 (61.6)	38 (25.2)	11 (7.3)	9 (6.0)	0.023
Mild (n=20)	8 (40.0)	7 (35.0)	4 (20.0)	1 (5.0)	
Moderate (n=28)	18 (64.3)	7 (25.0)	3 (10.7)	0 (0.0)	
Severe (n=11)	3 (27.3)	2 (18.2)	4 (36.4)	2 (18.2)	
Depression					
No (n=153)	89 (58.2)	41 (26.8)	15 (9.8)	8 (5.2)	0.042
Mild (n=19)	8 (42.1)	7 (36.8)	4 (21.1)	0 (0.0)	
Moderate (n=22)	18 (81.8)	3 (13.6)	0 (0.0)	1 (4.5)	
Severe (n=16)	7 (43.8)	3 (18.8)	3 (18.8)	3 (18.8)	
Anxiety					
No (n=152)	90 (59.2)	38 (25.0)	16 (10.5)	8 (5.3)	0.436
Mild (n=26)	10 (38.5)	10 (38.5)	4 (15.4)	2 (7.7)	
Moderate (n=26)	17 (65.4)	6 (23.1)	2 (7.7)	1 (3.8)	
Severe (n=6)	5 (83.3)	0 (0.0)	0 (0.0)	1 (16.7)	
Loss of appetite					
No (n=6)	6 (100)	0 (0.0)	0 (0.0)	0 (0.0)	0.011
Mild (n=28)	22 (78.6)	4 (14.3)	1 (3.6)	1 (3.6)	
Moderate (n=114)	59 (51.8)	37 (32.5)	13 (11.4)	5 (4.4)	
Severe (n=62)	35 (56.5)	13 (21.0)	8 (12.9)	6 (9.7)	
Best wellbeing					
No (n=4)	3 (75.0)	1 (25.0)	0 (0.0)	0 (0.0)	0.012
Mild (n=8)	5 (62.5)	2 (25.0)	0 (0.0)	1 (12.5)	
Moderate (n=74)	35 (47.3)	30 (40.5)	9 (12.2)	0 (0.0)	
Severe (n=124)	79 (63.7)	21 (16.9)	13 (10.5)	11 (8.9)	
Shortness of breath					
No (n=124)	67 (54.0)	35 (28.2)	14 (11.3)	8 (6.5)	0.629
Mild (n=8)	4 (50.0)	4 (50.0)	0 (0.0)	0 (0.0)	
Moderate (n=39)	26 (66.7)	8 (20.5)	4 (10.3)	1 (2.6)	
Severe (n=39)	25 (64.1)	7 (17.9)	4 (10.3)	3 (7.7)	
Constipation					
No (n=160)	100 (62.5)	33 (20.6)	17 (10.6)	10 (6.3)	0.149
Mild (n=22)	9 (40.9)	11 (50.0)	1 (4.5)	1 (4.5)	
Moderate (n=20)	10 (50.0)	7 (35.0)	3 (15.0)	0 (0.0)	
Severe (n=8)	3 (37.5)	3 (37.5)	1 (12.5)	1 (12.5)	

Patients who were admitted to the hospital three times or more were less likely than those who were admitted fewer times to be referred much too late to PC services, p=0.046. Also, patients who were not referred to long-term care or home health care were more likely to be referred to PC services much too late, p<0.001. Length of survival after first referral to PC services was much higher among patients with ideal referral time to PC services than others as being 373±174.9 compared to 16.4±35.3 among those whose referral time was described as much too late, p<0.001 (Table 5).

**Table 5:** Association between quality of care and quality of life with palliative care referral time in the patients with advanced cancer. *Chi-square test; **ANOVA

Variables	Timing of palliative care referral				P-value*
	Much too late	Too late	Appropriate	Ideal	
	N=122(%)	N=54(%)	N=22(%)	N=12(%)	
Frequency of hospital admission					
0-2 (n=84)	57 (67.8)	13 (15.5)	9 (10.7)	5 (6.0)	0.046
≥3 (n=126)	65 (51.6)	41 (32.5)	13 (10.3)	7 (5.6)	
Frequency of emergency room admission					
0-2 (n=97)	57 (58.8)	26 (26.8)	8 (8.2)	6 (6.2)	0.796
≥3 (n=113)	65 (57.5)	28 (24.8)	14 (12.4)	6 (5.3)	
Referral to long-term care					
No (n=196)	119 (60.7)	50 (25.5)	19 (9.7)	8 (4.1)	<0.001
Yes (n=14)	3 (21.4)	4 (28.6)	3 (21.4)	4 (28.6)	
Referral to home health care					
No (n=90)	71 (78.9)	14 (15.6)	4 (4.4)	1 (1.1)	<0.001
Yes (n=120)	51 (42.5)	40 (33.3)	18 (15.0)	11 (9.2)	
Referral to interdisciplinary team					
No (n=87)	51 (58.7)	23 (26.4)	9 (10.3)	4 (4.6)	0.948
Yes (n=123)	71 (57.7)	31 (25.2)	13 (10.6)	8 (6.5)	
Length of hospital stay (days)					
Mean±SD	23.0±25.1	21.7±22.2	15.5±16.2	21.9±23.7	0.617**
Length of survival after first referral to PC services					
Mean±SD	16.4±35.3	51.6±16.1	118.4±18	373±174.9	<0.001*
Do Not Resuscitate status before death					
Within 3 days (n=124)	72 (58.1)	30 (24.2)	14 (11.3)	8 (6.5)	0.418**
4-2 weeks (n=39)	27 (69.2)	10 (25.6)	1 (2.6)	1 (2.6)	
>2 weeks (n=47)	23 (48.9)	14 (29.8)	7 (14.9)	3 (6.4)	
Actively dying					
Yes (n=33)	25 (75.7)	5 (15.2)	2 (6.1)	1 (3.0)	0.170*
No (n=177)	97 (54.8)	49 (27.7)	20 (11.3)	11 (6.2)	
Reason of referral					
Transfer of care (n=97)	58 (59.8)	22 (22.7)	11 (11.3)	6 (6.2)	0.817*
Both of transfer of care and symptom management (n=113)	64 (56.7)	32 (28.3)	11 (9.7)	6 (5.3)	

In 28.8% of patients whose PPI ranged between 30% and 50% compared to 14.9% of those whose PPI ranged between 10% and 20% indicated too late referral time to PC, p=0.040. Other studied prognostic factors (PPS and ECOG) were not significantly associated with PC referral time (Table 6).

**Table 6:** Association between prognostic factors and palliative care time of referral among the patients with advanced cancer. *Chi-square test

Variables	Timing of palliative care referral				P-value*
	Much too late	Too late	Appropriate	Ideal	
	N=122 (%)	N=54 (%)	N=22 (%)	N=12 (%)	
Eastern Cooperative Oncology Group (ECOG)					
Group 3 (n=6)	3 (50.0)	2 (33.3)	1 (16.7)	0 (0.0)	0.852
Group 4 (n=204)	119 (58.3)	52 (25.5)	21 (10.3)	12 (5.9)	
Palliative Performance Status (PPS)					
10-20% (n=47)	35 (74.5)	7 (14.9)	3 (6.4)	2 (4.3)	0.040
30-50% (n=163)	87 (53.4)	47 (28.8)	19 (11.7)	10 (6.1)	
Palliative Prognostic Index (PPI)					
Group B (n=3)	2 (66.7)	0 (0.0)	1 (33.3)	0 (0.0)	0.473
Group C (n=207)	120 (58.0)	54 (26.1)	21 (10.1)	12 (5.8)	

## Discussion

Advances in cancer treatments observed in the last decades lead to remarkable survival benefits, and consequently the patients' quality of life [14]. Palliative care can play a fundamental role for early interventions regarding symptom palliation and offering psychosocial support that helped the survival and cancer patients' quality of life [3,5]. The present study was done to identify the timing of the first palliative consultation and referral of advanced cancer patients to the palliative care service in KFSHRC, Jeddah, Saudi Arabia.

Consultation of palliative care earlier is associated with lesser need for intensive medical care and enhanced quality outcomes for cancer patients [15]. In the current survey, more than half of the patients had a history of PC consultation. In them, PC consultation was described as late in 60.5% of the patients; a higher rate of late PC consultation has been reported in a cohort of cancer patients in Tennessee, USA (84.5%) [16]. In Brazil, cancer patients have been referred to palliative care late in the course of the disease [17].

In the current study, the mean length of survival time from referral to PC to death was 56.5 days and the median was 24 days. Different figures have been reported from different studies carried out in various countries. In Brazil [17], the mean time from entry into PC service to death was 72 days. In the USA [18], the median times were 42 and 57 days in two different studies. In Australia [19], a median time of 54 days has been reported. However, a very long median time (237 days) has also been observed in an American study [15]. A very close figure (median time = 21 days) has been reported in a study carried out in China [20]. In Lebanon [21], the overall median survival time was 18.7 months, time to first PC consultation was 17.9 months, and survival post-PC consultation was 0.6 months. The findings present in our study could be explained by the fact that our patients are mostly referred to palliative care too late.

Visiting ER more frequently (≥3 times) was associated with earlier PC consultation in this study compared to less frequent visiting (0-2 times). Also, patients who were referred to home healthcare were more likely to consult PC earlier compared to their counterparts. Also, in the current study, longer survival time was associated with earlier PC consultation. In accordance with others [15], PC consultation time was not associated with patient's demographic factors in the current survey.

The current survey revealed that none of the prognostic factors (ECOG, PPS, PPI) was significantly associated with PC consultation time while others have shown an association [22]. In a recent study carried out in Lebanon [21], late PC consultation was associated with a childless status, awareness of the diagnosis, and lack of palliative home care.

The present study revealed that referral to PC for both transfer of care and symptom management was associated with earlier PC. Delayed PC consultation was also observed in a recent study carried out in Lebanon [21].

Concerning the timing of palliative care referral among advanced cancer patients who sought consultation in the present study, it was too late and much too late in 25.7% and 58.1% of cases, respectively, whereas it was appropriate/ideal among only in 16.2% of them. In a recent study carried out by Barth et al., half of the patients were referred to the PC service within 3.7 months before death [23].

It has been documented that early integration of palliative care for patients with advanced cancer not only improves control of their physical symptom but also improves their psychological well-being [5]. Concerning presenting symptoms, patients with no pain, no affection of wellbeing, or loss of appetite were more likely than others to be referred for PC service much too late; those with severe nausea were less likely to be referred much too late to PC services; while patients with moderate depression also were most likely to be much too late referred to PC services.

Ideal or appropriate referral to PC service for patients with advanced cancer has also been associated with better quality of life by enhancing the patient's understands of their health problem and prospective prognosis and helping to cope and planning for advanced care [24]. In the current study, 60% of patients with advanced cancer experienced three or more hospital admissions in the last year of life compared to nearly 25% of patients in Canada who experienced two or more hospital admissions in the last month of life [25]. Both results indicate a need for improvement of the process of referral to PC services. Moreover, in the current study, patients who were admitted to the hospital three times or more were less likely than others to be much too late referred to PC services.

The present cohort revealed a mean of hospital stay for advanced cancer patients of 21.8±23.5 days, however, it was not associated with the timing of referral to PC service. Some others suggested that early PC referral is associated with fewer hospitalizations near the end of life [26].

In this study, almost 60% of the patients with advanced cancer were referred to the interdisciplinary team, mainly social workers. It has been reported that the integration of interdisciplinary PC services into the routine care of advanced cancer patients is highly recommended [27].

In the present study, the time interval between signing the DNR order and the patient’s death was three days in 59% of patients whereas it exceeded two weeks among 22.4% of them. In a study carried out in Korea, the median time between signing DNR consent to death was 1.76 days, which is a very short time. They attributed this finding to the fact that their study was conducted at a hospital in which patients tend to receive more active anti-cancer therapy [28].

Concerning the performance status, although in the present study, patients belonging to group 4 ECOG were more likely to consult PC than those of group 3, yet the difference was not significant, most probably due to the small number of patients in group 3, so results might be underpowered to detect a significant difference. Also, in the current survey, ECOG was not associated with the time of referral to PC. It has been confirmed from previous literature that the performance status of patients correlates positively with their response to treatment, survival, and quality of life [29]. It has been also observed that older patients with a good performance status have outcomes of treatment very similar to those of their younger counterparts [30].

Limitations and barriers

Illness-specific limitation includes lack of a clear transition between curative and palliative phases of management and prognostic uncertainty. Cultural limitation includes cultural and social barriers, a misconception that palliative care is only provided for patients with cancer near the end of their life and a lack of knowledge regarding the role played by the palliative care team. Oncologist (primary physician)-based limitation includes lack of knowledge regarding the potential role in palliative care services and misconception of them that palliative and hospice cares are the same. System-based limitation includes lack of clear guidelines concerning referral, limited resources for PC, not yet established in the study setting hospice care, advanced care planning and grief with bereavement program. Finally, the study-based limitation is that the study was carried out in a single-center, which could impact the generalizability of findings. Also, the retrospective nature of the study makes it subjected to some biases, including the incomplete recording of some patient-related factors and the possible exclusion of a few cancer patients admitted to other departments during the study period, and finally, there were difficulties in the collection of required data.

## Conclusions

The article tackles an important and rarely investigated subject in developing countries. A considerable proportion of terminal cancer patients, PC was consulted late, and the timing of PC referral was too late and much too late in most of those who consulted PC. Length of survival was higher among patients who reported early PC consultation and who with ideal referral time to PC services than others. Therefore, future considerations to facilitate early integration of PC in cancer patients' care is highly recommended through mainly improving staff education in communication skills and palliative care approach with established the advanced care planning and bereavement program. Patients should access PC services early to improve outcomes and quality of life. A further multi-centric study including patients from other facilities is highly recommended to be able to generalize the findings.

## References

[1] (2018). Palliative care. http://www.who.int/cancer/palliative/definition/en/.

[2] Hui D, Bruera E (2020). Models of palliative care delivery for patients with cancer. J Clin Oncol.

[3] Greer JA, Jackson VA, Meier DE, Temel JS (2013). Early integration of palliative care services with standard oncology care for patients with advanced cancer. CA Cancer J Clin.

[4] (2018). Integrating palliative care and symptom relief into primary health care: a WHO guide for planners, implementers and managers. https://apps.who.int/iris/bitstream/handle/10665/274559/9789241514477-eng.pdf.

[5] Temel JS, Greer JA, Muzikansky A (2010). Early palliative care for patients with metastatic non-small-cell lung cancer. N Engl J Med.

[6] Zimmermann C, Swami N, Krzyzanowska M (2014). Early palliative care for patients with advanced cancer: a cluster-randomised controlled trial. Lancet.

[7] Hui D, Kim SH, Roquemore J, Dev R, Chisholm G, Bruera E (2014). Impact of timing and setting of palliative care referral on quality of end-of-life care in cancer patients. Cancer.

[8] Smith TJ, Temin S, Alesi ER (2012). American Society of Clinical Oncology provisional clinical opinion: the integration of palliative care into standard oncology care. J Clin Oncol.

[9] Zimmermann C, Riechelmann R, Krzyzanowska M, Rodin G, Tannock I (2008). Effectiveness of specialized palliative care: a systematic review. JAMA.

[10] Hawley P (2017). Barriers to access to palliative care. Palliat Care.

[11] Hui D, Mori M, Watanabe SM (2016). Referral criteria for outpatient specialty palliative cancer care:an international consensus. Lancet Oncol.

[12] de Oliveira Valentino TC, Paiva BSR, de Oliveira MA, Hui D, Paiva CE (2018). Factors associated with palliative care referral among patients with advanced cancers: a retrospective analysis of a large Brazilian cohort. Support Care Cancer.

[13] Anwar S, Case A (2016). The art of prognostication in advanced cancer: missing the big picture. J Fam Med.

[14] Edwards BK, Noone AM, Mariotto AB (2014). Annual Report to the Nation on the status of cancer, 1975-2010, featuring prevalence of comorbidity and impact on survival among persons with lung, colorectal, breast, or prostate cancer. Cancer.

[15] Robbins SG, Hackstadt AJ, Martin S, Shinall MC Jr (2019). Implications of palliative care consultation timing among a cohort of hospice decedents. J Palliat Med.

[16] Osta BE, Palmer JL, Paraskevopoulos T (2008). Interval between first palliative care consult and death in patients diagnosed with advanced cancer at a comprehensive cancer center. J Palliat Med.

[17] Rozman LM, Campolina AG, López RVM, Kobayashi ST, Chiba T, de Soárez PC (2018). Early palliative care and its impact on end-of-life care for cancer patients in Brazil. J Palliat Med.

[18] Dalal S, Bruera S, Hui D (2016). Use of palliative care services in a tertiary cancer center. Oncologist.

[19] Good PD, Cavenagh J, Ravenscroft PJ (2004). Survival after enrollment in an Australian palliative care program. J Pain Symptom Manage.

[20] Gu X, Cheng W, Chen M, Liu M, Zhang Z (2016). Timing of referral to inpatient palliative care services for advanced cancer patients and earlier referral predictors in mainland China. Palliat Support Care.

[21] Bakouny Z, Assi T, El Rassy E (2019). Factors associated with the time to first palliative care consultation in Lebanese cancer patients. Support Care Cancer.

[22] Kao CY, Hung YS, Wang HM (2014). Combination of initial palliative prognostic index and score change provides a better prognostic value for terminally ill cancer patients: a six-year observational cohort study. J Pain Symptom Manage.

[23] Barth C, Colombet I, Montheil V (2020). First referral to an integrated onco-palliative care program: a retrospective analysis of its timing. BMC Palliat Care.

[24] Bagcivan G, Dionne-Odom JN, Frost J (2018). What happens during early outpatient palliative care consultations for persons with newly diagnosed advanced cancer? A qualitative analysis of provider documentation. Palliat Med.

[25] (2018). Palliative and end-of-life care. http://www.systemperformance.ca/report/palliative-end-of-life-care/.

[26] Greer JA, Pirl WF, Jackson VA (2012). Effect of early palliative care on chemotherapy use and end-of-life care in patients with metastatic non-small-cell lung cancer. J Clin Oncol.

[27] Ferrell BR, Temel JS, Temin S (2017). Integration of palliative care into standard oncology care: American Society of Clinical Oncology Clinical Practice Guideline update. J Clin Oncol.

[28] Baek SK, Chang HJ, Byun JM, Han JJ, Heo DS (2017). The association between end-of-life care and the time interval between provision of a do-not-resuscitate consent and death in cancer patients in Korea. Cancer Res Treat.

[29] Cella D, Eton D, Hensing TA, Masters GA, Parasuraman B (2008). Relationship between symptom change, objective tumor measurements, and performance status during chemotherapy for advanced lung cancer. Clin Lung Cancer.

[30] Garg P, Rana F, Gupta R, Buzaianu EM, Guthrie TH (2009). Predictors of toxicity and toxicity profile of adjuvant chemotherapy in elderly breast cancer patients. Breast J.

